# Is perioperative pro-B-type natriuretic peptide a good tool to evaluate surgical risk in cardiac surgery?

**DOI:** 10.1186/1749-8090-10-S1-A128

**Published:** 2015-12-16

**Authors:** Marta Matamala, Neslim Gálvez, Elisa Ochoa, Ana Barral, Javier Fañanás, Jose María Vallejo, Manuel Vázquez, Fernando Sorribas, Carlos Ballester

**Affiliations:** 1 Left a Servicio de Cirugía Cardiovascular, Hospital Universitario Miguel Servet, Zaragoza, Spain

## Background/Introduction

Predicting major adverse events and death in patients undergoing cardiac surgery is based on clinical risk scores such as Euroscore. This score has a moderate power for discriminating morbidity. Natriuretic peptides are well-established biomarkers in numerous clinical settings, prognostic, diagnostic and treatment of cardiac failure. In cardiac surgical patients the role of natriuretic peptides as risk markers is less well delineated.

## Aims/Objectives

To assess the utility of natriuretic peptides as risk markers in cardiac surgery

## Method

This study is a prospective longitudinal study of consecutive 135 patients undergoing on-pump cardiac surgery between 2012 and 2013. We evaluated preoperative euroSCORE II, preoperative and 24 h postoperative pro-BNP. The endpoints were: heart failure, renal failure, all-cause mortality at 12 months. Independent sample t-Test were performed.

## Results

One hundred thirty-five patients were available for analysis. the mean EuroSCORE II was 2,49%. Fourteen patients (10%) experienced postoperative heart failure and 30 patients (22%) renal failure. Within 12 months after surgery, six patients died. The "t" test showed significant augmentation of preoperative and postoperative pro-BNP in relation to heart and kidney failure.

## Discussion/Conclusion

Increased perioperative pro-BNP concentrations are associated with more incidence of postoperative heart failure and renal insufficiency. Elevated preoperative pro-BNP is not correlated with mortality. Postoperative pro-BNP adds little to the value of preoperative pro-BNP measurement alone.

